# Exosomes: the future of acellular nanotherapeutics in regenerative vascularization

**DOI:** 10.3389/fbioe.2025.1607605

**Published:** 2025-09-04

**Authors:** Jazzmyn S. Dawes, Maryam Abdelaal, Mary E. Landmesser, Mohammad Hossein Asgardoon, Olivia P. Waldron, Ji Ho Park, Neekita Jikaria, Dino J. Ravnic

**Affiliations:** Penn State Milton S. Hershey Medical Center, Hershey, PA, United States

**Keywords:** exosomes, regenerative, vascularization, stem cells, angiogenesis, wound healing

## Abstract

**Background:**

Ischemic disorders represent the world’s leading cause of morbidity and mortality and can emanate from pathology in both the macrovasculature and microvasculature. Current treatment options for macrovascular disease include surgical bypass, endovascular intervention, thrombolytic drugs, and pharmacologics (vasodilators). However, when ischemia occurs at the microvascular level, conventional vascular surgical approaches are typically not feasible. In this setting, complex reconstructive surgery may be warranted, especially if concurrent open wounds are present. Thus, new pro-angiogenic treatment strategies that facilitate microvascular regenerative vascularization and wound repair are welcome.

**Methods:**

We present a comprehensive overview of both stem cell-derived and mature–cell-derived exosomes in the context of regenerative vascularization and wound repair, focusing on cargo mechanisms and biomaterial delivery strategies. We also highlight how materials science will be instrumental to both therapeutic delivery and development of fully acellular pro-angiogenic bioengineered exosomes. All cited studies involving exosomes complied with the International Society of Extracellular Vesicles guidelines. To assess the clinical relevance and gaps, we visited clinicaltrials.gov, where keywords “exosome” and “vascular” were searched. Other parameters such as completion status, country, and exosome type further refined our search.

**Results:**

Exosomes were found to promote angiogenesis and improved wound healing outcomes primarily *via* Vascular Endothelial Growth Factor, FGF2, miR-126, Wnt/β-catenin, Notch and PI3K/Akt pathways. Clinicaltrials.gov revealed only 3 out of 15 completed human exosome studies worldwide related to regenerative vascularization.

**Conclusion:**

Therapies utilizing exosomes as an acellular approach to regenerative vascularization are promising, though challenges with scalability remain. Further mechanistic understanding, standardization, and controlled trials are compulsory prior to widespread human application.

## 1 Introduction

Ischemia is characterized by insufficient blood flow to an area of the body, resulting in the restriction of oxygen and nutrient transfer. Ischemic disorders are the leading cause of death affecting various organs and triggering a diverse range of pathologies such as stroke (cerebral ischemia), heart attack (myocardial ischemia), chronic wounds (peripheral ischemia), and mesenteric ischemia (Chambers et al., 2021; Darwin and Tomic-Canic, 2018; Farber and Eberhardt, 2016). Current treatment options for large-vessel (macrovascular) arterial inflow problems include surgical bypass, endovascular intervention, thrombolytic drugs, and pharmacologics (vasodilators) Chambers et al., 2021; Seront et al., 2024). Unfortunately, these treatment modalities, especially the surgical options, are not effective in addressing ischemic changes emanating from the microvasculature. Even the most modern surgical advances only allow for the manipulation of vessels down to the ∼0.5 mm (500 µm) diameter range, as evidenced by the recent development of super-microsurgical techniques (Badash et al., 2018). As the proper microvasculature is generally defined as vessels less than 200 µm in diameter any direct manipulation of arterioles, capillaries, and venules is still precluded (Kaul and Ito, 2004).

According to Feuer et al. “microvascular dysfunction has been defined as a varied set of conditions which includes vessel destruction, abnormal vasoreactivity, *in situ* thrombosis, and fibrosis that can ultimately result in tissue ischemia manifesting as tissue damage and progressive organ failure” (Feuer et al., 2022). One of the most dramatic examples of microvascular dysfunction is seen with coronary microvascular angina and infarction. Here, patients have symptoms related to decreased blood flow to the heart muscle but often with normal angiographic findings (Geltman et al., 1990). A multitude of studies have demonstrated that there is little benefit to any pharmacologic treatment options (Marinescu et al., 2015; Bairey Merz et al., 2020). Another clinical example where microvascular impairment exists are lower extremity wounds with or without the presence of diabetes (Chao and Cheing, 2009). A 10-year study of over 120,000 veterans showed that microvascular disease was associated with a 3.7-fold increased risk of amputation; and when occurring alongside macrovascular disease the risk further increased to 22.7-fold (Beckman et al., 2019). Most frustrating, is that some patients who undergo macrovascular peripheral arterial repair, *via* stent or bypass, still progress to limb amputation (Behroozian and Beckman, 2020). For example, a retrospective study showed that 59% of 22 postsurgical revascularizations still needed amputation for foot ischemia despite bypass graft patency after a 14-month follow-up in a cohort of patients also suffering from end-stage renal disease (Johnson et al., 1995). As microvascular disease extends from the arteriole level down through the capillary bed newer approaches are desperately needed to treat ischemia originating here (Behroozian and Beckman, 2020).

Currently, reconstructive surgery allows for the transfer of remote tissue and its functional microvasculature to an ischemic site following any needed optimization of macrovascular arterial inflow (Coriddi et al., 2022). However, like approaches used to treat ischemia originating from the macrovasculature, flap surgery itself cannot completely mitigate all microvascular ischemia at the recipient site. Thus, new pro-angiogenic treatment strategies that facilitate regenerative vascularization and wound repair are warranted.

Angiogenesis, the development of new blood vessels from preexisting vasculature, represents the body’s natural response to hypoxic tissue ischemia (Adair and Montani, 2010). Angiogenesis is required to maintain tissue perfusion in the setting of physiologic changes, such as injury (Kalka et al., 2000), and an imbalance of angiogenesis is regarded as a main contributor of delayed wound healing (Brem and Tomic-Canic, 2007; Roy et al., 2009). Without proper neovascularization, acute injuries can become chronic. Chronic wounds are frequently seen in patients dealing with diabetes, advanced age, or tobacco use (Kalka et al., 2000; Si et al., 2019; Sen, 2021). In these patients, disturbances in either the macro- and/or micro-vasculature results in impaired transport of oxygen and nutrients to the afflicted tissues. This can further be appreciated in a variety of clinical dilemmas.

Specifically, diabetic foot ulcers (DFU) can arise from a combination of trauma, peripheral neuropathy, and both macro- and micro-vascular disease (Wang et al., 2022b). The annual incidence of DFU is approximately 18.6 million globally (Zhang et al., 2020) and they are associated with significant morbidity. Approximately 20% of patients require hospitalization and/or a major (above the ankle) or minor (limited to the foot) lower extremity amputation (Hicks et al., 2018; Lipsky et al., 2012). A recent systemic review and meta-analysis by Chen *et al.* reported a 5-year mortality rate of 50.9%, with cardiovascular disease and infection being the most common causes (Chen et al., 2023a). This is comparable or greater than the 5-year mortality of common cancers such as breast (9%), lung (72.3%), and colorectal cancer (64.4%) (NIH, 2020). The estimated financial impact on the U.S. healthcare system is between $9 and $13 billion annually (Sen, 2019). The mainstay of treatment includes lifestyle change, infection control, wound debridement, and localized care with order of importance being determined by individual patient need (Alexiadou and Doupis, 2012; Everett and Mathioudakis, 2018). These strategies are largely aimed in preventing the wound from enlarging as true healing is seldom achieved in the setting of uncorrectable microvascular disease.

There exists only one FDA-approved DFU treatment option, Regranex, a Platelet-derived growth factor (PDGF) gel that has been shown to induce angiogenesis and microvascular expansion. The initial study evaluated more ​than 350 diabetic patients who had chronic wounds of at least 8 weeks’ duration. It was reported that treatment with Regranex (becaplermin gel), significantly increased the percentage of patients with completely healed wounds compared to placebo (Wieman et al., 1998). However, these results were often not reproducible, including in animal models (Chan et al., 2006) preventing widespread clinical implementation. These limitations and inconsistent results have sparked the development of alternative modalities, including wound dressings. Niezgoda et al. evaluated DFU wound healing in over 70 patients treated with OASIS Wound Matrix, an acellular wound care product, compared to Regranex. They found that after 12 weeks of treatment, 18 (49%) OASIS-treated patients had complete wound closure compared with 10 (28%) Regranex-treated patients, highlighting an alternative, cost effective wound dressing (Niezgoda et al., 2005). A newer group of studies by McLaughlin et al. highlight the healing effects of naltrexone (NTX), an opioid receptor antagonist, in comparison to Regranex within preclinical rodent models. The authors demonstrate that topical application of NTX accelerated wound closure 5-fold, through increased cell proliferation, angiogenic factor secretion (e.g., VEGF, PDGF, FGF-2 and a-SMA) and microvascular formation (McLaughlin et al., 2017; McLaughlin et al., 2013). Despite critical demand for pro-angiogenic wound healing therapeutics, there has been a relative paucity in treatment modalities that can stimulate microvascular formation to alleviate local tissue ischemia (Wang et al., 2019; Sen, 2021). The interplay of pro- and anti-angiogenic growth and signaling molecules with surrounding modulatory cell types (e.g., endothelial cells, fibroblasts, and macrophages) challenges clinicians and researchers to develop new multifaceted therapeutic strategies (Zhang et al., 2024).

The field of regenerative medicine has increasingly utilized stem cell-based therapies to address ischemia and enhance wound healing, primarily through immune modulation and promotion of angiogenesis. These cells possess self-renewal capacity and can differentiate into multiple cell types, offering various strategies to stimulate neovascularization. Among these, mesenchymal stem cells (MSCs) derived from bone marrow or adipose tissue have been the most studied. Typically, MSCs are applied directly to wounds or delivered *via* hydrogel-based carriers to improve their viability and therapeutic effect. However, despite their promise, stem cell therapies face challenges such as immune rejection, limited cell survival without appropriate storage, and complex handling requirements. These limitations underscore the need for acellular, pro-angiogenic therapies, such as exosomes.

## 2 Exosomes: nanoscale modulators

**FIGURE 1:**
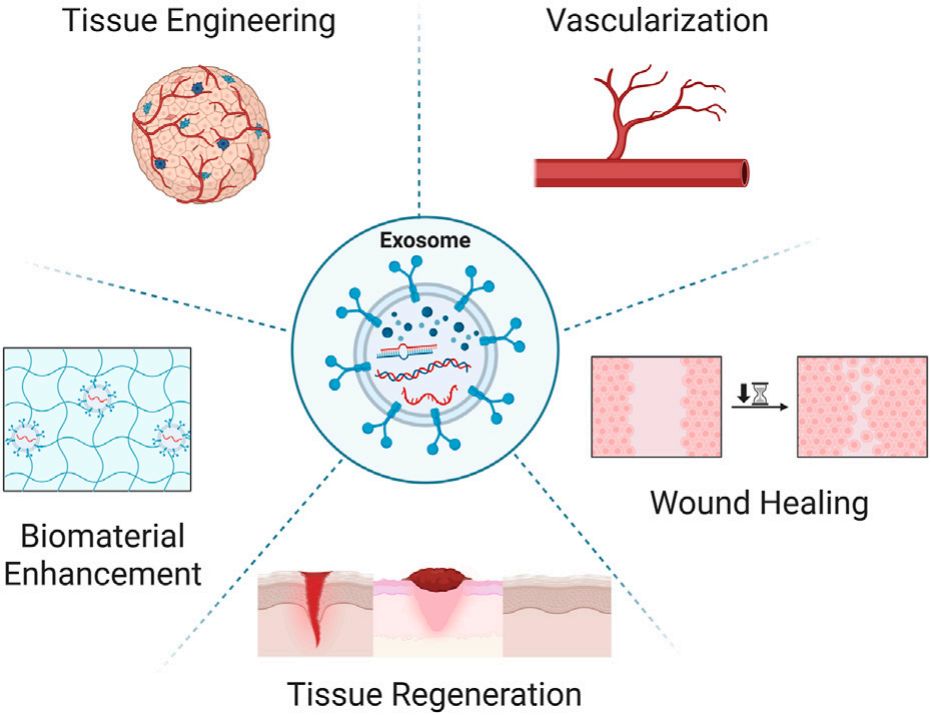
Exosome applications in regenerative medicine. Exosomes, a small class of extracellular vesicles have headlined the regenerative research and medical fields as a novel multifunctional therapeutic. Exosomes have been found to be instrumental in various regenerative hallmarks including tissue engineering, inducible vascularization, accelerated wound healing, tissue regeneration, and enhanced biomaterial efficacy. Created in BioRender. Ravnic, D. (2025) https://BioRender.com/mbrx7wr.

Acellular exosomes have pro-angiogenic and pro-healing qualities similar to stem cells without the risk of immune rejection. Specifically, they demonstrate potential as a regenerative nanotherapeutic with high biocompatibility, stability, uncomplicated storage requirements, homing effects, and controllable dosing (Couch et al., 2021; Chen et al., 2021; Luan et al., 2017; Dong et al., 2023). Exosome delivery also overcomes challenges faced by conventional pharmacologics, as their nanoscale size allows for passage through vascular barriers (e.g., blood-brain barrier) and deep tissue penetration even in the setting of existing ischemia. Exosomes can be sourced from virtually any bodily fluid, including blood, urine, saliva, breast milk, cerebrospinal fluid, semen, and *in vitro* cell culture supernatant. Following isolation, they retain functionality within a range of physiological processes including immune response, tissue repair, and angiogenesis. Diverse cell surface receptors and embedded cargo can modulate wide-reaching biological processes (Figure 1) (Dilsiz, 2022). Given these qualities, it is postulated that exosomes can be utilized in gene transfer, cell-cell communication, and drug delivery. This review highlights the potential of exosomes for regenerative vascularization at the microvascular level.

### 2.1 Biogenesis

**FIGURE 2:**
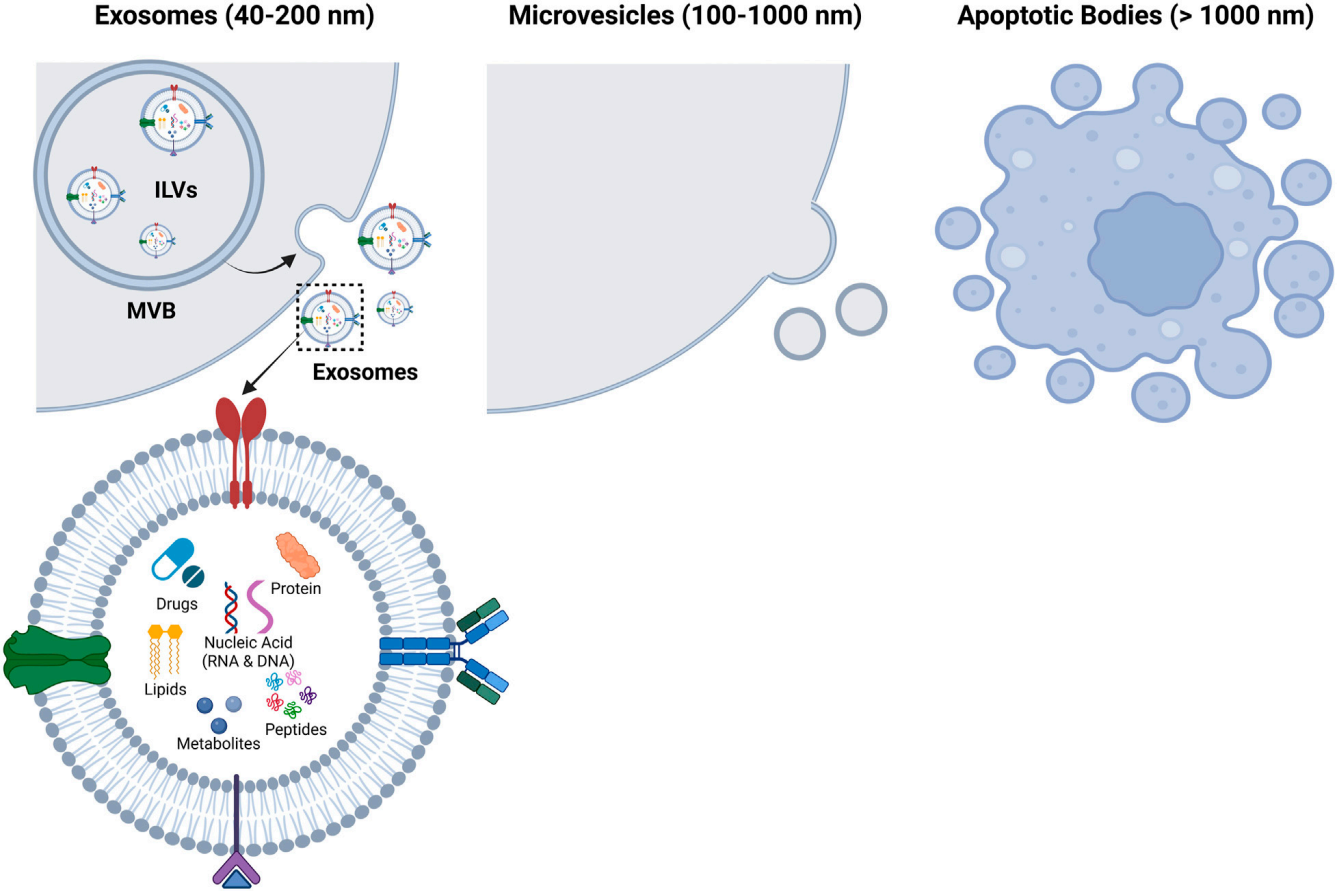
Exosome biogenesis. The three main classes of extracellular vesicles include: exosomes, microvesicles, and apoptotic bodies. Exosome biogenesis is hallmarked by endosomal invagination resulting in the formation of multi-vesicular bodies (MVBs) which contain intraluminal vesicles (ILVs). The membranes of MVBs eventually fuse with the plasma membrane, which allows for the ILVs to be released from the cell as exosomes. This biogenesis process equips the resulting exosome populations with heterogenous surface markers and encapsulated bioactive cargo. Microvesicles originate from plasma membrane budding, while apoptotic bodies are a result of programmed cell death blebbing. Created in BioRender. Ravnic, D. (2025) https://BioRender.com/mbrx7wr.

Exosomes are a class of small extracellular vesicle (sEV) with a diameter range of 40–200 nm released from all cell types that have a notable secretome. Other types of EVs include microvesicles (MVs) ranging in size from 100 to 1,000 nm, and apoptotic bodies, which are greater than 1,000 nm in diameter (Couch et al., 2021). MVs are generated through plasma membrane budding, while apoptotic bodies are formulated during programmed cell death blebbing (Gurung et al., 2021). Exosomes, which is the focus of this review, are formed through endosomal invagination followed by exocytosis (Figure 2).

Briefly, endosomes are formed by cell membrane invagination where bioactive molecules on the cell membrane or lumen accumulate within the early-sorting endosomes. Late-sorting endosomes converge, forming multivesicular bodies (MVB) after a second invagination event (Rädler et al., 2023). Then the MVBs fuse with the plasma membrane to release the bioactive exosomes (Couch et al., 2021; Ren, 2019). Biogenesis is co-coordinated by the four primary, highly conserved proteins of endosomal sorting complex required for transport (ESCRT) with key steps mediated by ESCRT-0, ESCRT-I, ESCRT-II, and ESCRT-III proteins and other chaperones. ESCRT-0 and ALIX help sort ubiquitinated molecules (cargo) into the endosome, ESCRT-I and -II coordinate the luminal budding of intraluminal vesicles (ILVs), and ESCRT-III filaments assist with scission of ILVs. ILVs in the endolysosomal pathway are degraded after formation while ILVs of precursor exosomes are expelled from the parent cell after formation (Frankel and Audhya, 2018; Bissig and Gruenberg, 2014).

### 2.2 Cargo formation and characterization

Most cargo within exosomes is acquired through encapsulation of membrane and cytosolic molecules tagged for ubiquitination. Glycoproteins have also been found sorted into exosomes *via* N-linked glycosylation (Liang et al., 2014). Likewise, non-ubiquitinated proteins can be sorted into MVBs through Cos proteins, which provide a Ub-signal in *trans*, sorting non-ubiquitinated MVB cargo into the ESCRT- and Ub-dependent pathway (Ren, 2019; MacDonald et al., 2015). Cos proteins are highly ubiquitinated and accumulate into subdomains on the endosomal membrane, where they provide the Ub-sorting signals in *trans* to cargo proteins they associate with immobilizing target cargo within endosomes prior to being sorted into ILVs (MacDonald et al., 2015). Following biogenesis and release, exosomes travel to a target site where they are taken up by cells and release their cargo to either initiate or suppress biological activity (Dixson et al., 2023). Exosomes primarily enter and release their internal (luminal) cargo to target cells through endocytosis (micropinocytosis) or cell membrane fusion while their external (coronal) content is delivered to target cells *via* receptor-ligand interactions. The coalescence of diverse external and internal modulatory cargo stimulates cascade responses below the membrane surface, highlighting exosomes as a multifunctional delivery platform for intercellular communication and bioactive components (Todorova et al., 2017; Chen et al., 2021; Lee et al., 2024).

Although the International Society of Extracellular Vesicles (ISEV) has outlined specific criteria to accurately distinguish exosomes from other vesicle types, they are still a highly heterogeneous population (Kalluri and LeBleu, 2020) carrying membrane receptors unique to their parent cell, such as MHC Class I and II molecules (Gurung et al., 2021). In addition to parental markers, proteins associated with vesicle trafficking and biogenesis like tetraspanins (CD81, CD9, and CD63), specific stress proteins (HSP70, HSP90), ESCRT proteins (TSG101, ALIX), membrane fusion proteins (Rabs, ARF6), and signaling proteins are considered to be exosome specific (Todorova et al., 2017; Tan et al., 2024). Exosomes also encapsulate genetic cargo such as DNA, miRNA, mRNA, and lncRNA, protecting it from enzymatic degradation (Todorova et al., 2017; Ren, 2019). Heterogeneity is a direct reflection of distinct exosomal characteristics related to size, cell of origin, luminal cargo, coronal cargo, and functional modulation of target cells (Kalluri and LeBleu, 2020).

## 3 Exosomes in angiogenesis and wound repair

Angiogenesis is the process of post-natal vascular growth where new blood vessels sprout from existing ones, providing nourishment to tissues and organs. The process is dependent upon the activation of endothelial cells (ECs) in response to angiogenic stimuli such as growth factors (e.g., Vascular Endothelial Growth Factor (VEGF) and Basic Fibroblast Growth Factor (bFGF)), cytokines, hypoxia, and mechanical cues. This primarily occurs *via* sprouting or intussusceptive angiogenesis (Adair and Montani, 2010). Intussusceptive angiogenesis is defined as the splitting of pre-existing, perfused blood vessels by intraluminal tissue pillars that form through endothelial invaginations that eventually divide the vascular lumen (Wong et al., 2020). Although it is theorized complete vascular restoration involves both forms of angiogenesis, vessel sprouting, is the better characterized, classical process used to stimulate neo-microvascular formation within avascular tissues such as those affected by hypoxia or injury (Bixel et al., 2024; Dudley and Griffioen, 2023; Fang et al., 2023). In adults, sprouting angiogenesis is predominantly triggered by hypoxia, which is characterized by the imbalance of tissue O_2_ homeostasis (Semenza, 2010). Sprouting angiogenesis is described by the activation, proliferation, migration and remodeling of ECs. Once activated, ECs sprout from the parent vessel toward the angiogenic stimulus. Matrix Metalloproteinases (MMPs), a class of proteolytic enzymes, assist with EC migration and invasion into surrounding tissue by degrading the extracellular matrix (ECM), clearing a path for EC tip cells (Stojkovic, 2024). This tightly regulated process is instrumental to wound healing across anatomic sites (Zhang et al., 2019; Huang et al., 2021; Bian et al., 2019; Carter et al., 2022) and is mediated by molecular signals (e.g., VEGF, PDGF, TGF-β, FGF, ANG, TIE, HIF, NOTCH, and WNT) and the coordinated cross-communication of fibroblasts, pericytes, and immune cells (Carter et al., 2022; Guo et al., 2024; Carmeliet and Jain, 2011). The equilibrium between pro- and anti-angiogenic signals collectively modulate the process (Carmeliet and Jain, 2011). The master angiogenic regulator, VEGF, interacts with its corresponding receptors (VEGFR1/2/3) to trigger EC proliferation and microvascular development (Todorova et al., 2017; Baruah and Wary, 2019; Carter et al., 2022). For example, molecules such as Flk-1 (VEGFR2) and Ang-1, a key regulator of vessel integrity, were found to be essential in promoting tube formation and maturation *in vitro* and *in vivo* (Thirunavukkarasu et al., 2022; Zhao et al., 2025; Liang et al., 2016). In contrast, when the anti-angiogenic gene, Vash1, is downregulated, *in vitro* angiogenesis is enhanced (Liang et al., 2016). Angiogenic remodeling and expansion of the microvasculature, serves as a pivotal step in the progression of wound healing.

The coordinated wound healing phenomena has four overlapping phases including hemostasis, inflammation, proliferation (angiogenesis), and remodeling, with each stage being governed by distinct cell types and regulated by multiple signaling pathways. Macrophages (Mφs), fibroblasts, pericytes, and platelets are instrumental across all stages and their interactions with ECs are temporally and spatially regulated. These cellular interactions take place concurrently in an extracellular matrix (ECM) that is undergoing dynamic remodeling to initiate new microvascular growth from the established vasculature. This process starts within 4 days following injury and can be seen clinically by the presence of granulation tissue. This new, pink, soft tissue is the beginning of wound repair as new capillaries composed of integrin-activated ECs, fibroblasts, and Mφs move into the fibrin and fibronectin-rich environment. Generally, Mφs secrete angiogenic factors, fibroblasts support new ECM development, and platelets release supportive growth factors, all interacting with the residual microvasculature to expand oxygen and nutrient delivery to the healing wound (Tonnesen et al., 2000). When these processes are suboptimal, ischemia can ensue, followed by the wound unable to heal. At the cellular level, many nonhealing ischemic wounds are stalled in the inflammatory phase, unable to progress to normal angiogenesis. Many diverse modalities, including cell therapies, have been attempted to remedy this but stem cells may have the most potential to induce therapeutic angiogenesis (Si et al., 2019).

Stem cell and adult cell-derived exosomes have been found to modulate angiogenesis through a diverse cargo load including bioactive proteins, mRNA, and small non-coding RNAs (Hussain et al., 2022; Wang et al., 2020b; Yuan et al., 2019; Ren, 2019) making them potentially suitable for therapeutic application (Baruah and Wary, 2019). For example, an early study demonstrated *in vitro* that exosomes rich in Tspan8-CD49d enhance EC proliferation, sprouting, and migration to enhance mature tube formation (Olejarz et al., 2020; Nazarenko et al., 2010). The formation of mature, perfusable tubes is a critical step in the final stages of angiogenesis, and the larger reparative process of wound repair (Huang et al., 2025). Researchers have utilized a variety of stem cells and adult cell-derived exosomes to initiate microvascular development and subsequent wound repair (Moeinabadi-Bidgoli et al., 2023; Xu et al., 2025).

## 4 Stem cells and their exosomes

Stem cells (SC) are broadly characterized by their ability to self-renew and differentiate, making them ideal for regenerative purposes (Si et al., 2019; Wang et al., 2024a). However, their only widespread use clinically has been for the treatment of osteoarthritis, autoimmune disorders and bloodborne malignancies, such as leukemia and lymphoma (Kirkeby et al., 2025; Hoh, 2025). Because success often relies on a matched donor, translation to other pathologies has been somewhat limited. If allogenic cells are used, they are likely to suffer from immune-mediated rejection. Although research is ongoing, there are differences among stem cell classes regarding potential for regenerative vascularization that need to be taken in the context of ethical concerns and practicality. Fortunately, stem cells can also exert their effects in a paracrine fashion (Tan et al., 2024; Baruah and Wary, 2019) suggesting that exosomes derived from SCs may have the potential to be a powerful acellular therapeutic not subject to immune rejection or ethical limitations (Table 1) (Hussen et al., 2024).

**TABLE 1:** Regenerative effects of stem cell-derived exosomes. Regenerative outcomes and mechanism(s) of action of ESC, iPSC, BMDC, ASC, and EPC-derived exosomes are presented within the context of regenerative vascularization.

Stem cells and their exosomes					
Origin cell (exosome type)	Embryonic stem cell (ESC)	Induced pluripotent stem cell (IPSC)	Bone marrow-derived stem cell (BMDC)	Adipose-derived Stem Cells (ASC)	Endothelial progenitor cell (EPC)
Regenerative Outcome	Accelerated wound closure; enhanced angiogenesis	Increased angiogenesis and enhanced vascular regeneration	Induction of cell proliferation, migration, and angiogenesis; Reduction of inflammation; enhanced wound healing	Increased induction of proangiogenic activity; acceleration of wound healing; re-epithelialization and collagen deposition	Promotes endothelial regeneration via enhanced proliferation and migration of Ecs; accelerated wound healing
Mechanism(s) of Action	EC senescent recovered via Nrf2 activation FGF2 signaling TGF-β receptor 2 signaling	Promotes viability of epidermal cells, ECs and fibroblasts	DMOG-induced stabilization of HIF1-α activates AKT/mTOR pathway Increased M2 Mφ polarization circMYO9B delivery	Activation of Wnt/β-catenin pathway miR-144-3p/NFE2L2/HIF1α signaling Nrf2 Pathway SIRT1 activation	Increased expression of FGF-1, eNOS, IL-8, ANG-1, E-selectin, VEGFA, VEGFR-2, CXCL-16 Erk1/2 or p53 signaling pathways Mesenchymal-endothelial transition activation
Advantage	Pluripotent potential	Does not require embryonic destruction	Ubiquitous	Easily accessible and ubiquitous; Multifunctional	Multifunctional
Disadvantage	Sourcing Ethical Concerns	Difficult to manufacture; possible risk of teratoma	Need to access cortical bone resulting in painful isolation process	Limited clinical data	Limited in availability due to difficulty isolating cells
Reference	Chen et al., 2019; Bae et al., 2018; Pang et al., 2021	Hu et al., 2015; Ye et al., 2019	Liang et al., 2019; Wang et al., 2024b; Liu et al., 2020	Wu et al., 2021; Hu et al., 2023; Li et al., 2023a;	Li et al., 2016b; Li et al., 2016c; Li et al., 2018, Hu et al., 2019b; Hu et al., 2019a; Xu et al., 2020; Ke et al., 2017

### 4.1 Embryonic stem cells

Embryonic stem cells (ESCs) are isolated from the inner cell mass of mammalian blastocysts and can be grown indefinitely in culture under correct conditions (Takahashi and Yamanaka, 2006). Their pluripotency offers the ability to treat diseases originating from any of the three germ layers: ectoderm, mesoderm, and endoderm, and their exosomes have been studied for the repair of mesoderm-origin cardiovascular pathologies. Chen *et al.* found that human ESC exosomes (ESC-Exo) helped heal pressure ulcers by opposing endothelial senescence through activation of Nrf2, a proangiogenic mediator (Chen et al., 2019). Similarly, one group showed that specific ESC-Exo microRNA accelerated skin healing *via* the TGF-β receptor 2 pathway (Bae et al., 2018). Others have demonstrated that ESC-Exo can promote myocardial angiogenesis and repair *via* FGF2 signaling (Pang et al., 2021). Unfortunately, even with their significant therapeutic potential, ESC-Exo are still curtailed by ethical concerns regarding ESC sourcing (Takahashi and Yamanaka, 2006), leading researchers to investigate other sources for cellular pluripotency.

### 4.2 Induced pluripotent stem cells

In 2006 Takahashi and Yamanaka used somatic cell reprograming *via* four transcription factors, Oct4, Sox2, Klf4, and c-Myc, to create an induced pluripotent stem cell (iPSC) (Puri and Nagy, 2011). iPSCs hold therapeutic potential similar to ESCs without embryonic destruction, obviating ethical concerns. This has made iPSCs and their exosomes (iPSC-Exo) a promising target in regenerative medicine and angiogenesis.

Hu *et al.* showed that iPSC-Exo induced angiogenesis and improved recovery in an ischemic hindlimb rodent model (Hu et al., 2015). Another group used iPSC-EC Exo as a pro-angiogenic agent and demonstrated the importance of exosomal miR-199b-5p (Ye et al., 2019). Others used an aortic ring model to show that iPSC-Exo enhanced vascular regeneration in old mice. The authors also suggest that iPSC-Exo could induce the formation of phenotypically and functionally younger exosomes within older mice. This was further corroborated when iPSC-Exo increased tube formation ability and uptake capacity of acetylated low-density lipoprotein within injured ECs, replicating the beneficial effects provided by young mouse exosomes (Li et al., 2023b).

To illustrate translational potential, a report (Gao et al., 2020) demonstrated the benefits of iPSC-to-adult-cell exosomes in ameliorating myocardial ischemia (MI) outcomes in a porcine model. Another animal study delineated the effects of autologous vs. allogenic iPSC-Exo for the promotion of wound healing (Lu et al., 2019). The authors found that wound capillary density was increased after autologous iPSC-Exo treatment compared to allogenic and sham controls. It was also reported that both autologous and allogenic iPSC-Exo were effective in promoting the viability of epidermal cells, ECs and fibroblasts, the cellular amalgam vital for wound healing. Together, these studies demonstrate that iPSC-Exo may be useful for regenerative vascularization and tissue repair.

Because iPSCs are laborious and costly to produce, and have the potential for teratoma formation, clinical translation has been quite limited. Theoretically, these downsides also extend to any of their exosomes. This has led to the investigation of multipotent stem cells that generate mesodermal repair, are harvested easily, and do not carry the risk of teratoma formation.

### 4.3 Mesenchymal stem cells

**FIGURE 3:**
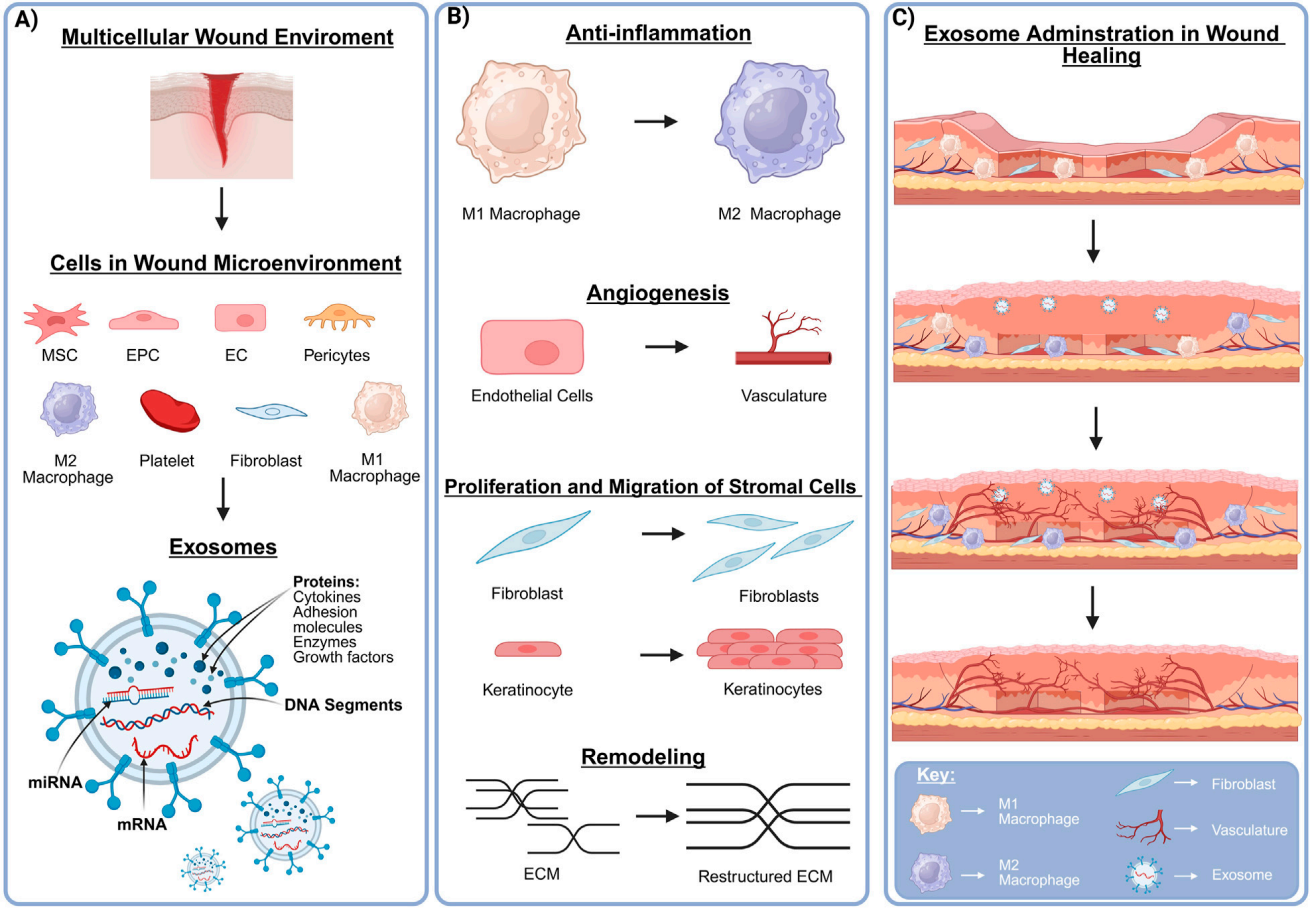
Exosomes and the multicellular wound microenvironment. Proper wound healing is dependent on interdependent multicellular communication within the local microenvironment. **(A)** The cellular milieu within a healing wound includes local and recruited modulators. These cell types secrete exosomes with diverse cargo including pro-angiogenic and pro-healing cytokines, growth factors, nucleic acids, and adhesion molecules. **(B)** These exosomes exert significant therapeutic potential in wound repair through facilitating macrophage polarization, microvascular expansion, re-epithelialization, and modulation of the remodeled ECM. **(C)** An extensive range of studies have demonstrated that exosomes are a useful acellular tool in enhancing the wound healing process *via* immune cell modulation and increased vascularization. Created in BioRender. Ravnic, D. (2025) https://BioRender.com/mbrx7wr.

Mesenchymal stem cells (MSCs), a subset of mesenchymal stromal cells, are multipotent adult stem cells that can self-renew and differentiate, albeit in a more limited capacity. However, because they can be easily retrieved and do not require reprogramming, they have an advantage over germ-layer specific treatment. The utilization of mesenchymal stem cells for mesodermal repair, including regenerative vascularization, has found widespread interest. MSCs can be isolated from most tissues including adipose and bone marrow (Si et al., 2019; Lin et al., 2010), however, use for regenerative vascularization is limited by cell quantity and in allogenic use, the possibility of immune rejection. Bypassing these limitations, their exosomes retain bioactive angiogenic functionality similar to the parent cell (Figure 3).

Studies have shown the pro-angiogenic wound healing potential of MSC exosomes (MSC-Exo) *via* different pathways including the Akt/eNOS and PTEN/PI3K/Akt pathways (Yu et al., 2020; Qiu et al., 2020; Li et al., 2016c). While others have reported promotion of angiogenesis and accelerated wound healing through activating the hnRNPU/CBL/KDM1A/VEGFA pathway *via* circMYO9B delivery (Wang et al., 2024b). Additionally, exosomes have been found to activate various Wnt/β-catenin signaling pathways, which are necessary in regulating both developmental and therapeutic angiogenesis (He et al., 2020; Dejana and Kühl, 2010). In a rat skin burn model, one group found that MSC-Exo expressing Wnt4 induced translocation of β-catenin into recipient cells, promoting angiogenesis (Zhang et al., 2015).

Notably, MSC-Exo were found to help not only trigger regenerative vascularization, but the induced microvasculature was functional under physiologic conditions in some studies. Vascular functionality after MSC-Exo treatment has been assessed using laser blood flow analysis in an ischemic murine model (Komaki et al., 2017) while others have demonstrated similar tube formation abilities *in vitro* and *in vivo* (Hu et al., 2015; Wang et al., 2022a). This suggests the potential capacity for clinical translation with varied underlying mechanisms of action. Irrespective of MSC sourcing, the pro-angiogenic potential of MSC-Exo is considered ubiquitous. For example, exosomes derived from dental pulp stem cells have cell-homing abilities that aid in tissue repair through enhancing cell proliferation and migration (Ganesh et al., 2022). However, not all sources of MSC are appropriate for clinical translation, due to limitations in quantity and ease of retrieval leading to two primary MSC subtype exosomes being used for regenerative vascularization: bone marrow derived stem cells (BMSC) and adipose stem cells (ASC).

#### 4.3.1 Bone marrow-derived stem cells

BMSCs are clonogenic fibroblast-shaped cells with the ability to differentiate across multiple mesodermal lineages including osteoblasts, chondrocytes, adipocytes, and hematopoiesis-supporting stroma (Derubeis and Cancedda, 2004). Exosomes released from these cells (BMSC-Exo) have demonstrated the capacity to induce cell proliferation and angiogenesis in wound repair. BMSC-Exo pre-conditioned with dimethyloxaloylglycine (DMOG), an angiogenic small molecule that stabilizes HIF-1α, were reported to augment tube formation, migration and regenerative capabilities through activation of the AKT/mTOR pathway (Liang et al., 2019). Another group identified that BMSC-Exo carrying circMYO9B amplified EC proliferation and migration leading to angiogenesis (Wang et al., 2024b). Melatonin-stimulated BMSC-Exo were able to significantly improve diabetic wound healing by inhibiting inflammation through increased M2 Mφ polarization, promoting angiogenesis, and collagen synthesis *in vivo* (Liu et al., 2020). While BMSCs exist in large quantities, their isolation is painful as the cortical bone needs to be breached for isolation and retrieval. This has ushered in the era of SC retrieval from more facile sites, such as subcutaneous tissue.

#### 4.3.2 Adipose-derived stem cells

ASCs are a subset of MSCs that are primarily housed in subcutaneous adipose tissue, making them easily accessible and ubiquitous. Exosomes from these cells have been found contributing to angiogenic and healing *events in vitro and in vivo*. One group reported that overexpression of exosomal mmu_circ_0001052 promoted proliferation, migration and angiogenesis in human umbilical cord endothelial cells (HUVECs) (Liang et al., 2022). In the same way, ASC-derived exosomes (ASC-Exo) were seen to exert increased induction of pro-angiogenic activity through EC migration and angiogenesis compared to BMSC-derived exosomes (Pomatto et al., 2021). He *et al.* created a skin lesion model through hydrogen peroxide (H_2_O_2_) exposure on human keratinocytes (HaCaT) and dermal fibroblasts (HDF) to assess the wound healing potential of ASC-Exo (He et al., 2020). They found that exosome treatment exponentially enhanced the proliferation and migration of HaCaT and HDFs while inhibiting apoptosis after H_2_O_2_ treatment. Overall, they conclude that the wound healing effects provided by ASC-Exo are mediated by encapsulated MALAT1, a transcriptional regulator of cell migration/motility, that targets miR-124 and activates the Wnt/β-catenin pathway. Wu and associates stimulated ASCs with LPS, a common component of pathogenic wounds, to assess the effects of ASC-Exo on angiogenesis *in vitro* (Wu et al., 2021). The study demonstrated increased production of angiogenic molecules such as IL-8, with significantly increased activation of CREB, AP-1, and NF-κB signal transduction pathways following exosome treatment. An investigation into the cardio-protective effects of ASC-Exo in a mouse MI model reported significant microvascular expansion, angiogenesis, and inhibition of cardiomyocyte apoptosis *via* miRNA-205 (Wang et al., 2023). ASC-Exo have also been integrated into hydrogel scaffolds, accelerating wound healing and angiogenic affects through activation of miR-144-3p/NFE2L2/HIF1α signaling (Hu et al., 2023). The Li group found that ASC-Exo containing miR-138 mediated hastened wound closure, increased reepithelization, had faster collagen deposition rates, and increased blood vessel formation through activation of SIRT1, a silent wound healing modulator (Li et al., 2023a). Lastly, ASC-Exo promote wound healing through vessel formation *via* Nrf2, a cytoprotective transcription factor. Specifically, this study conducted by Li *et al.* utilized a DFU model to show that ASC-Exo utilize Nrf2 to enhance angiogenic activity and subsequent wound healing by activating endothelial progenitor cells (Pomatto et al., 2021; Li et al., 2018). Collectively, these data suggest that ASC-Exo hold considerable utilization value in ischemic wound repair.

### 4.4 Endothelial progenitor cells

Endothelial progenitor cells (EPCs) are unipotent circulating cells that express markers similar to mature ECs but can also participate in new vessel formation. Thus, they have shown promise for regenerative vascularization and tissue repair in various models including MI, stroke, peripheral arterial disease, and wound healing (Li et al., 2016c; Wagner et al., 2018). In addition to neovascularization, EPCs participate in pro-angiogenic activities by promoting endothelial regeneration through enhanced proliferation and migration of endogenic ECs *via* paracrine mechanisms (Cun et al., 2022). Although their potential for regenerative vascularization is widely appreciated, their classification as “true” stem cells is highly debated. EPCs express classical biomarkers of immature stem cells like CD133 while also displaying mature cell markers such as CD34, CD31, VEGFR-2, von Willebrand factor (vWF), CD144, Tie2, CD117, CD62E and CD45 (Wagner et al., 2018; Zhao et al., 2016). Investigators have reported that CD133^+^/VEGFR-2^+^ cells reflect immature progenitor cells localized mainly in the bone marrow, while AC133^−^/CD34^+^/VEGFR-2^+^ cells represent mature stem cells with limited proliferative capacity. In actuality, many propose EPCs to be a bridge between “true” stem cells whose primary function is to regenerate and repair damaged tissues through differentiation and “mature” stem cells that regulate blood vessel formation in ischemic areas (Zhao et al., 2016). Indisputably, with both characteristics, these vascular stem cells seem an ideal platform upon which to build therapeutic efforts for regenerative vascularization, especially in the context of wound repair (Li et al., 2016c; Li et al., 2021). Like other cells, EPCs modulatory effects are passed down *via* paracrine secretomes, with various reports demonstrating pro-angiogenic properties during tissue regeneration and repair across both the macrovasculature and microvasculature (Peters, 2018).

For example, three separate investigations have shown that EPC exosomes (EPC-Exo) promoted macrovascular repair and hastened reendothelialization in a rat model of ballon-induced vascular injury by augmenting EC function (Li et al., 2016b; Hu et al., 2019b; Hu et al., 2019a). Outside of their direct effects on macrovascular EC function they have also shown therapeutic effects *via* increased expression of angiogenic molecules such as fibroblast growth factor 1 (FGF-1), nitric oxide synthase (eNOS), interleukin-8 (IL-8), angiopoietin-1 (ANG-1), E-selectin, VEGF-A, VEGFR-2, and chemokine ligand-16 (CXCL-16) (Li et al., 2016b; Li et al., 2016c). The effects of these pro-angiogenic molecules are synergized with ECs concurrently expressing depressed levels of anti-angiogenic molecules like matrix metalloprotein-9 (MMP-9) following EPC-Exo treatment (Li et al., 2016c). These profound effects suggest potential application for the microvascular repair and expansion required in healing tissues.

Unsurprisingly, multiple studies have shown that EPC-Exo can accelerate wound healing by stimulating angiogenesis through varied signaling pathways, such as Erk1/2 or p53 (Li et al., 2018; Xu et al., 2020). When *in vitro* wound healing and tube formation assays were performed using EPC-Exo -treated epithelial cells and ECs there was a significantly strengthened pro-angiogenic effect (Li et al., 2016b; Li et al., 2016c), notably on the proliferation, migration, and angiogenic capacity of HUVECs (Li et al., 2016b). Another group showed that EPC-Exo can also affect both angiogenesis and healing *via* activation of the mesenchymal-endothelial transition (Ke et al., 2017). *In vivo* wound healing studies have demonstrated that EPC-Exo concentration correlates with microvascular density and subsequent tissue repair (Li et al., 2018). The culmination of these *in vitro* and *in vivo* findings collectively supports the theory that EPC-Exo can drive regenerative vascularization and tissue repair in a variety of settings. However, like other SCs, EPCs are difficult to isolate and are limited in quantity. This has led many to seek alternative cell sourcing for exosome-based therapeutics, namely, mature cells.

## 5 Mature cells and their exosomes

The complex process of microvascular expansion and wound healing is a revolving interrelated network of cellular and molecular events. This reemphasizes the necessary communication between regenerative stem cells and their relationship with the surrounding mature cells, such as Mφs, fibroblasts, ECs, pericytes and even acellular platelets. Exosomes can exert diverse effects on cell-to-cell or cell-to-microenvironment communication, irrespective of cell maturity (Heo, 2022; Jella et al., 2018). Here, we present the effects of mature cell-derived exosomes in regenerative vascularization and tissue repair (Figure 3; Table 2).

**TABLE 2:** Regenerative effects of mature cell-derived exosomes. Regenerative outcomes and mechanism(s) of action of EC, pericyte, Mφ, fibroblast, and platelet-derived exosomes are presented within the context of regenerative vascularization.

Mature cells and their exosomes					
Origin cell (exosome type)	Endothelial Cell (EC)	Pericyte	Macrophage (Mφ)	Fibroblast	Platelet
Regenerative Outcome	Microvascular expansion	Functional recovery of vasculature and microvascular sprouting	Inducible M2 Mφ polarization. Increased EC migration, proliferation, and tube formation. Increased collagen deposition and re-epithelialization	Controls vessel segment length and branching by promoting EC proliferation, migration and angiogenic tubule formation	Increased cell migration, proliferation, and angiogenesis promoting wound repair
Mechanism(s) of Action	Tip cell induction via DLL4:Notch signaling Enhanced EC-EC communication Inhibitition of EC senescence via ATM repression miR-214-mediated communication and ATM repression	PC-EC coordinate restored by cPWWP2A Enhanced angiogenic sprouting EC function restored	Delivery of proangiogenic factors (e.g., VEGF, Wnt3a, miR-130a) Downregulation of E2F2	Delivery of angiogenic factors (e.g., ANGPT-1, ANGPT-2, Tie-2) Delivery of dual-functioning angiogenic/mitogenic molecules (e.g., IL-1α, IL-4, IL-8/CXCL8, GRO-α, GRO-β and GRO-γ)	AKT and ERK pathways Crosstalk between TGF-β and YAP Delivery of miR-126 resulting in increased expression of VEGF, bFGF, and TGF-β1
Advantage	Exo carry pro-angiogenic factors	Spatiotemporally released pro-angiogenic factors	Multifunctional	Promotes vessel formation, maturation, and stabilization	Multifunctional
Disadvantage	Limited vascularization data	Limited in availability due to difficulty isolating cells	N/A	Limited data	Limited data
Reference	Sheldon et al., 2010; Ju et al., 2014; Todorova et al., 2017; Jia and Sowers, 2020; Van Balkom et al., 2013	Liu et al., 2019; Limanjaya et al., 2019; Yuan et al., 2019	Kim et al., 2019; Gangadaran et al., 2020; Huang et al., 2021, Hussain et al., 2022; Yang et al., 2021	Han et al., 2021; Xie et al., 2020; Hu et al., 2021	Hayon et al., 2012; Torreggiani et al., 2014; Sun et al., 2019

### 5.1 Endothelial cells

ECs are mature cells that line both the macro- and micro-vasculatures. They are essential to maintaining normal homeostasis and are heavily recruited following injury to grow the microvasculature in response to heightened tissue needs of oxygen and nutrients (Yuan et al., 2022; Xie et al., 2020). Angiogenesis is a complex process that mandates EC migration, proliferation, tube formation and stabilization followed by functional microvascular restoration (Baruah and Wary, 2019). Studies have shown that ischemic wounds resulting from either macrovascular or microvascular dysfunction suffer from dampened EC quantity and EC cluster quality, notable components of a proper angiogenic response (Roy et al., 2009). Subsequent tissue repair is dependent on normal angiogenesis maintained by constant cross-cellular communication emanating from ECs. This makes EC exosomes (EC-Exo) a potential avenue for therapeutic exploitation through a variety of underlying mechanisms.

The effects of EC-Exo may be attributed to specific angiogenic signaling molecules within their cargo. It is been suggested EC-Exo mediated transfer of delta-like 4 (DLL4), inhibited notch signaling which resulted in the tip cell phenotype induction of ECs, an integral step in initiating microvascular expansion (Sheldon et al., 2010). Another group found that EC-Exo effects are correlated to syndecan-4/syntenin signaling *via* angiopoietin-2 (Ju et al., 2014). EC-Exo have also been noted to carry a variety of potent complementary pro-angiogenic factors including VEGF, TGFβ, bFGF, MMP2, and MMP9 (Baruah and Wary, 2019). EC-Exo also carry an abundance of relevant miRs (e.g., miR-126 and miR-19a-3p) which are transported to target ECs and improve vascularization through EC-EC communication (Todorova et al., 2017; Jia and Sowers, 2020). Others report that miR-214-mediated communication triggers angiogenesis by repressing ATM expression and preventing recipient EC senescence using both *in vitro* and *in vivo* models (van Balkom et al., 2013). Thus, it has become clear that both genomic and non-genomic cargoes in EC-Exo are potentially capable of inducing regenerative vascularization. Even with this evidence, microvascular growth and subsequent tissue repair require the support of other mature cells and their exosomes.

### 5.2 Pericytes

Angiogenesis depends on the coordinated interaction of EC and their supporting cell, the pericyte (PC), that is embedded within the vascular basement membrane (Li et al., 2018). While ECs expand the microvasculature during angiogenesis, maturation is not possible without the paracrine signaling and structural support provided by pericytes. In general, PCs are vital to normal microvascular function and upkeep; and do so by maintaining a synergistic relationship with a variety of neighboring cells. By detecting changes in the local microenvironment, PCs can change the molecules they secrete to modulate communication with nearby cells to regulate angiogenesis and tissue regeneration, often intermediated by inflammation (Sharma et al., 2022).

Fundamentally, the regenerative paracrine effects of pericytes are suggestibly due to their exosomes (PC-Exo). PC-Exo contain a variety of pro-angiogenic factors such as VEGF, CTGF, PLGF, FGF, microRNA, and circRNA whose release depends on the surrounding milieu (Sharma et al., 2022). Ye *et al.* found that hypoxia upregulated circEhmt1 expression in PC-Exo during hyperglycemia and that exosomal modulation might protect ECs in chronic diabetic wounds (Ye et al., 2021). Another study showed that high glucose-treated PCs secreted exosomes with cPWWP2A, another circular RNA hypothesized to be vital in the proper coordination of PC and EC function during tube formation *in vitro* (Liu et al., 2019). *In vivo*, others have described that PC-Exo enhanced microvascular sprouting from an aortic ring (Limanjaya et al., 2019). One notable translatable experiment showed that PC-Exo could rescue EC functionality with regards to blood flow regulation and oxygen delivery in mice recovering from spinal cord injuries (Yuan et al., 2019). Unfortunately, despite these benefits, pericytes are rare, and isolation is difficult, hindering any potential exosome clinical translation.

### 5.3 Macrophages

Macrophages (Mφs) are a class of myeloid leukocytes equipped with phagocytic activity and inflammatory signaling properties making them central to tissue defense and homeostasis. They express pathogen associated molecular pattern receptors (PAMPs) and damage associated molecular pattern receptors (DAMPs) that detect the presence of signature molecules that report infection or tissue damage (Carlson et al., 2009). Precursor Mφs, or monocytes, are often recruited from the blood to replace long-term resident Mφs (Liu et al., 2021). It is known that Mφs presence in tissue is a dynamic process governed by changes in the local microenvironment (Carlson et al., 2009), though augmented during tissue repair. During normal wound repair, there is a transition from the initial inflammatory phase to the proliferative phase. This transition is a critical regulatory point that dictates overall healing outcome (Kim et al., 2019; Klar et al., 2018). Mφs are integral to this transition with different subtypes having unique functions.

The most described Mφ subtypes relevant to inflammation and tissue repair are classically activated M1 Mφs with pro-inflammatory functions and activated M2 Mφs which are anti-inflammatory and pro-regenerative (Bian et al., 2019; Chen et al., 2023b; Abdik et al., 2024; Wang et al., 2020a; Kim et al., 2019; Wang et al., 2024b). Furthermore, there can be a phenotypic switch from M1 to M2 at specific time points as required by the dynamic repair process (Kim et al., 2019). Generally, M1 Mφs are detected immediately following injury and M2 Mφs later-on as the tissue is being repaired (Chen et al., 2023b; Gangadaran et al., 2020; Wang et al., 2020a; Njock et al., 2015). Expectedly, M2 Mφs promote requisite angiogenesis and ECM synthesis/remodeling (Heo, 2022; Liu et al., 2021; Mills and Ley, 2014). The mechanisms that facilitate each process are governed by numerous pro-angiogenic molecules including VEGF, TNFα, IL-8, and bFGF (Liu et al., 2021; Butoi et al., 2016; Osinsky et al., 2011).

Like many other cell types, Mφs secrete exosomes that reflect their own features to help generate a favorable healing microenvironment (Hazrati et al., 2022). It has been demonstrated that exosomes derived from M2 Mφs promote cutaneous wound healing by inciting *in situ* conversion of M1 Mφs into a reprogrammed M2-like phenotype (Kim et al., 2019). Apart from polarization, it has been explored whether Mφ-exosomes (Mφ-Exo) can affect adjacent endothelial cell activity. Studies support this and suggest the Mφ-Exo have pro-angiogenic properties, especially when influenced by miRNA modification. Mφ-Exo have been shown to contain more VEGF, Wnt3a, and miR-130a than their parent cell leading to augmented formation of blood vessels *in vivo* (Gangadaran et al., 2020). Another study demonstrated that M2 Mφ-Exo have increased levels of both miR-155-5p and miR-221-5p that enhance EC migration, proliferation and tube formation *via* downregulation of the anti-angiogenic molecule E2F2 (Huang et al., 2021; Hussain et al., 2022; Yang et al., 2021). Finally, it has been reported that when M2 Mφ-Exo were injected into a skin wound, enhanced angiogenesis, collagen deposition and re-epithelialization was seen, promoting healing (Lyu et al., 2022). While ECs, pericytes, and Mφs work together *via* exosome crosstalk to create a proper healing environment, these cells are supported by the vital fibroblast.

### 5.4 Fibroblasts

Dermal fibroblasts (DFs) are localized to the dermis of the skin and are essential for skin remodeling and maturation during tissue repair. These cells are responsible for building the ECM through a variety of collagens and cytokines, and fibroblast exosomes have been found to be important for augmenting microvascular density in the newly formed ECM (Han et al., 2021). It has also been reported that fibroblast exosomes contribute to microvascular architecture by controlling vessel segment length and branching. This study also showed that mechanical stress-induced fibroblast exosomes significantly increased EC proliferation, migration, and angiogenic tubule formation (Xie et al., 2020). Another study utilizing 3D-SFnws-adhering human dermal fibroblasts (HDFs), reported that released exosomes carried higher levels of multiple angiogenic factors including ANGPT-1, ANGPT-2, and Tie-2, in addition to dual-functioning angiogenic/mitogenic molecules like IL-1α, IL-4, IL-8/CXCL8, GRO-α, GRO-β and GRO-γ (Hu et al., 2021). These data suggest that fibroblast-derived exosomes may be applicable for ECM deposition but also regenerative vascularization during the tissue repair process.

## 6 Acellular-derived exosomes

### 6.1 Platelets

Platelets are anuclear cell fragments which accumulate at sites of vascular injury which upon activation release a variety of bioactive substances to recruit other cells. First, additionally recruited platelets work to form a clot inhibiting blood loss from the injured site. Then other cells are recruited, including Mφs, to begin the tissue repair phase of wound healing (Brill et al., 2005). Like with nucleated cells, platelets have the ability to release pro-angiogenic exosomes.

Hayon *et al.* showed that in cerebral ischemia, platelet exosomes carried a variety of growth factors related to central angiogenic pathways such as AKT and ERK, and had the ability to increase compensatory angiogenesis (Hayon et al., 2012). Torreggiani *et al.* showed that platelet-derived exosomes improved cell proliferation, migration, and angiogenesis, based on crosstalk between TGF-β and yes-associated protein (YAP) to promote wound healing (Torreggiani et al., 2014). Another group assessed the effects of platelet exosomes carrying miR-126, a known pro-angiogenic transcript. They described that exosomes promoted EC proliferation and migration through the overexpression of miR-126 and angiogenic factors like VEGF, bFGF, and TGF-β1 (Sun et al., 2019). Overall, further research on platelet-exosomes appears a worthwhile goal for promoting regenerative vascularization therapeutics.

## 7 Preclinical applications of exosomes in regenerative vascularization

Neovascularization is critical to proper wound healing (Yuan et al., 2022). Unfortunately, efforts to promote therapeutic angiogenesis have not found widespread clinical success. It is difficult to therapeutically initiate and subsequently stabilize the microvasculature, which under normal settings is contingent on a dynamic orchestration of angiogenic factors (Wagner et al., 2018). There has yet to be a single therapy that can replicate this exceedingly complex process. While exosomes can release a multitude of angiogenic factors concurrently, they suffer from rapid bloodstream clearance (Ren, 2019). Therefore, it is vital to develop exosome delivery platforms that can maintain their functionality at the target site as required. Biomaterials and exosome engineering methods are being called upon to fill this need.

### 7.1 Exosome-embedded hydrogels

Clinically translatable biomaterials need to be biocompatible, biodegradable, receptive to microvascular ingrowth from the recipient, and have low immunogenicity (Si et al., 2019; Eke et al., 2017). Over the past two decades hydrogels have found increasing clinical use as they meet these criteria. They are widely used for dermal repair and serve as a platform for advanced surgical reconstruction. They are a hydrophilic biomaterial that can be further refined for embedded drug and cell delivery (Wang et al., 2019; Wu et al., 2016; Lokhande et al., 2018). These attributes have made them especially appealing for regenerative vascularization effects.

Researchers have found that hydrogels offer maximal angiogenic bioactivity when loaded with cells (e.g., ECs, MSCs) or growth factors (e.g., VEGF) (Wu et al., 2016; Fujita et al., 2004; Awada et al., 2014; Eke et al., 2017; da Silva et al., 2019). These enhanced hydrogels have had some success in achieving sustained growth factor release compared to bolus delivery (Lokhande et al., 2018; da Silva et al., 2019). This has manifested a variety of hydrogels, composed of collagen, gelatin (denatured collagen), fibrin, or hyaluronic acid, embedded with various growth factors. While embedded growth factors are preferred to embedded cells, as the latter are prone to cell necrosis and immune rejection (Chambers et al., 2021), they are not ideal because the spatiotemporal release, especially of multiple growth factors, cannot be easily controlled.

**FIGURE 4:**
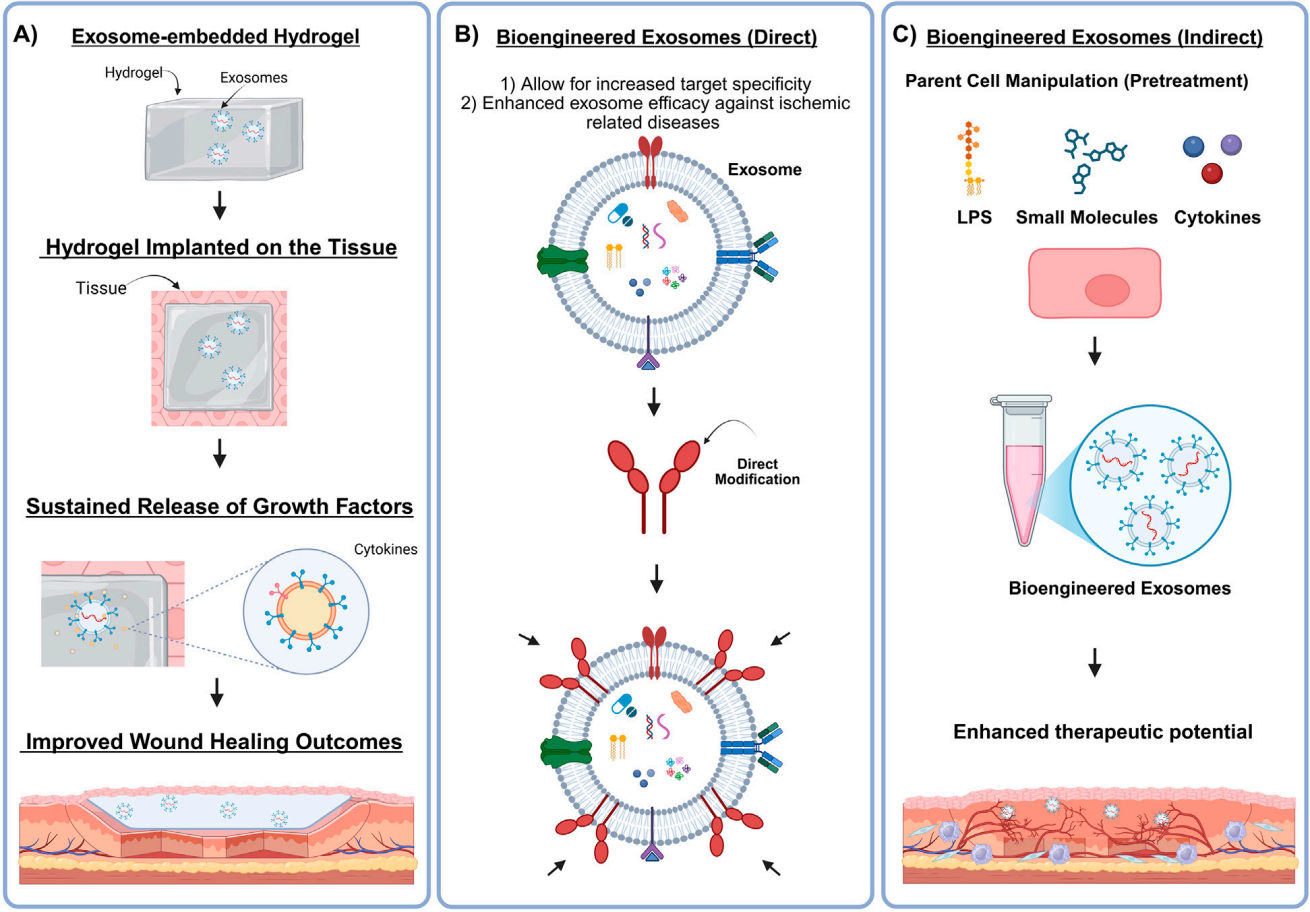
Preclinical applications of exosomes in regenerative medicine. **(A)** Exosomes have been found to enhance hydrogel efficacy within wound healing through sustained release of pro-angiogenic growth factors and cytokines. **(B and C)** Direct and indirect methods of exosome engineering have allowed researchers to enhance target specificity while also augmenting the exosomes regenerative capacity through surface alterations or parent cell priming. Created in BioRender. Ravnic, D. (2025) https://BioRender.com/mbrx7wr.

Most recently the fields of medicine and materials science have paired up to overcome this ongoing limitation to develop exosome loaded bioactive hydrogel scaffolds (Peshkova et al., 2022). Exosome embedded hydrogels offer controlled release of multiple angiogenic growth factors and molecules, disease-specific scaffold customization, and local delivery enhancement (Tan et al., 2024; Awada et al., 2014; Wilgus, 2008) (Figure 4A). This approach may offer more clinical potency as an ischemia remedy with recent studies showing more effective microvascular reestablishment compared to single growth factor utilization (Awada et al., 2014; Wilgus, 2008). Hydrogel customization (e.g., thermosensitive, photosensitive, pH-responsive) offers even further refinements for controlling exosome bioactivity and sustainability within the wound site (Dong et al., 2023; Wang et al., 2019). One review highlights hydrogel properties including crosslink density, pore size, and degradation rate, explaining their significant impact on the release kinetics of exosomes characterized by a rapid onset followed by a slower steady release phase (Fan et al., 2024). Wu and associates report enhanced exosome-embedded hydrogel efficacy with expedited wound healing *via* increased cell proliferation and angiogenesis (Wu et al., 2016). The designers of the study comment that exosome release from the FEP scaffold dressing was evaluated by a micro bicinchoninic acid (BCA) protein test kit and reported to be sustainably released for up to 3 weeks, while maintaining biological function. In a diabetic wound model, Li *et al.* found that a HAP/chitosan-hydrogel loaded with exosomes enhanced bioactivity, induced angiogenesis, and expedited healing (Li et al., 2016a). These biological results are supplemented by the fact that these exosomes were stably released within medium for 2 days, with TEM imaging reporting that some retain their original structures after 6 days. Most exciting is that even some conventional surgical implants (e.g., cochlear, intraocular, contraceptive, *etc.*) could be enhanced by the local release of exosomes and their bioactive attributes (Tan et al., 2024). However, not all surgical problems require an implanted scaffold. Therefore, newer approaches are being developed that can offer exosome bioactivity without large material implantation.

### 7.2 Bioengineered exosomes

Many groups have displayed the effects of bioengineered exosomes in hopes of intensifying the potential therapeutic impact of nanoparticle delivery of pro-angiogenic and tissue repair molecules. The development of bioengineered exosomes is making strides within the research community with the promise of bioactive modulation with less reliance on initial cellular sourcing (Dilsiz, 2022; Choi et al., 2021). Therefore, several exosome bioengineering methods have been developed to expand the utilization of exosomes in regenerative medicine in hopes of enhancing their efficacy in therapeutic angiogenesis (Choi et al., 2021). Exosome bioengineering takes place *via* direct alteration following exosome biogenesis or indirect manipulation through the originating cell.

Direct manipulation of exosomes includes biochemical modification or bioengineering of exosome physical characteristics such as surface markers (Tan et al., 2024; Dong et al., 2023; Rädler et al., 2023) (Figure 4B). These methods can be broken down into pre-production cargo loading techniques (e.g., transfection, co-incubation, and electroporation) and post-production approaches (e.g., sonication, extrusion, freeze-thaw cycles, incubation, and hypotonic dialysis). The exact approaches are categorically dependent on whether they are utilized before or after exosome biogenesis. Exosome surface marker engineering allows for heightened cell and tissue specificity and represents a physical modification aspect of direct exosome engineering (Li et al., 2025; Antes et al., 2018).

Studies have shown that exosomes pretreated with various bioactive molecules exert distinctive effects with regards to cell-to-cell or cell-to-microenvironment communication (Heo, 2022; Li et al., 2025). In one impactful study, authors found that selenium, an element with known healing effects, augmented the therapeutic potential of MSC-derived exosomes. This study demonstrated that exosomes pre-treated with selenium had increased expression levels of pro-angiogenic genes (e.g., ANGPT1, KDR and VEGF) while anti-angiogenic genes such as VASH1 were downregulated. These results *in vitro* imply therapeutic potential through angiogenic gene regulation, demonstrated by active fibroblast migration and the enhanced production of pro-angiogenic secretory molecules required for wound healing (Heo, 2022; Park et al., 2018).

The indirect modification of exosomes includes the priming of the parent cell through pretreatment using inflammatory cytokines, angiogenic factors, or genetic modulators (RNAs) (Figure 4C) (Miceli et al., 2021). Researchers have found that the state of the host cell/tissue directly impacts the resulting exosome populations’ therapeutic potential. For example, overexpression of HIF-1α in dental pulp MSCs significantly enhanced exosome secretion and angiogenesis *in vivo* compared to control MSCs (Gonzalez-King et al., 2017). A newer investigation presented evidence that exosomes derived from MSCs that are preconditioned with Empagliflozin (EMPA), a sodium-glucose cotransporter inhibitor, facilitated enhanced diabetic cutaneous wound repair through stimulation of angiogenesis *via* PTEN/AKT/VEGF pathway (Wang et al., 2025). Others show that melatonin-stimulated MSC-Exo enhanced wound healing through the regulation of Mφs within the microenvironment (Liu et al., 2020). A similar investigation demonstrated that hypoxia-stimulated ASC-Exo had an enhanced therapeutic effect compared to normal exosomes (Hu et al., 2023). While many have reported the significant benefits of these modified exosomes, the ultimate goal of exosome engineering remains to maximize their valuable cargo and effect without unnecessary components to create a translatable pro-angiogenic modality that is optimizable and safe.

Even with these modifications, the applications of exosomes within the clinic setting have yet to overcome FDA proposed regulatory standards. Challenges in quality control, batch variability, mass producibility, limited clinical data and knowledge of underlying mechanism of action (MOA) have halted the clinical translation of exosomes in treatment of ischemia (Wang et al., 2024c). While these hurdles have limited the output of human-based exosome trials, some researchers have still undertaken the challenge. We utilized a U.S. database using keywords “exosome” and “vascular” to track the current clinical landscape of exosome-related trials, where only 60, worldwide, related to the vascular system. Clinicaltrials.gov highlighted that within these 60, a mere 15 have been completed. To supplement this feat, five of these studies were conducted in the United States, with many lacking results and complete understanding of underlying MOA. Within the 60 highlighted studies, only 3, dealt with regenerative vascularization (Table 3). These slim outlooks re-emphasize the critical need for enhanced understanding of exosome MOA, joined by more in-depth clinical evaluations of long term-effects and documented adherence to ISEV guidelines in hopes of establishing reproducible, good manufacturing practice (GMP)-compliant exosome therapeutic platforms.

**TABLE 3:** Worldwide exosome-related clinical trials. Clinicaltrials.gov revealed only 60 vascular dysfunction exosome-related trials have been launched worldwide. Out of these 60, only 15 have been completed, while only 3, denoted by ∗, are pertinent to regenerative vascularization. Out of these, two are utilized for diagnostic purposes, with only one being interventional.

Disorder	Exosome type	Status	Country	Outcome	Study title	NCT number
Diabetic Foot Ulcer (DFU)∗	Wharton’s Jelly-Derived MSC-Exosomes	PHASE1; Completed	Egypt	N/A	Efficacy and Safety of Wharton’s Jelly-Derived Mesenchymal Stem Cell Exosomes in the Treatment of Diabetic Foot Ulcers: A Double-blinded Randomized Controlled Clinical Trial	NCT06812637
Hemodynamic Instability; Autophagy	Blood or urine	N/A; Completed	Taiwan	N/A	A Study of Exosome Proteomics and Hemodynamics in Sepsis	NCT03267160
Stroke Rehabilitation∗	Serum	N/A; Completed	Italy	Exo are a potentially effective biomarker for post- ischemic stroke	Extracellular Vesicles as Stroke Biomarkers	NCT05370105
Prostate Cancer	Urine	N/A; Completed	United States	EPI can be reliably utilized to predict disease grade of prostate cancer	Clinical Evaluation of the ‘ExoDx Prostate IntelliScore’ (EPI)	NCT03031418
Hypertension	Urine	N/A; Completed	United States	N/A	When the Kidney Reacts to Nutritional Changes	NCT04142138
Hypertension	Urine	N/A; Completed	Switzerland	N/A	New Biomarkers and Difficult-to-treat Hypertension	NCT03034265
Port-Wine Stain	Blood	N/A; Completed	United States	N/A	Pathogenic Mechanisms of Port Wine Stain and Repository of Port Wine Stain Biopsy Samples	NCT02051101
Non-Small Cell Lung Cancer	Serum	N/A; Completed	Belgium	N/A	Detection of Circulating Biomarkers of Immunogenic Cell Death (ICD)	NCT02921854
Gastroesophageal Reflux Disease; Barrett’s Esophagus	Blood	N/A; Completed	United States	N/A	Reflux-Induced Oxidative Stress in Barrett’s Esophagus: Response, Repair, and Epithelial-Mesenchymal-Transition	NCT02579460
Uveitis; Vasculitis; Ocular Inflammatory Disease	Vitreous, anterior chamber fluid, whole blood	N/A; Completed N/A; Completed	United States	N/A	The Vitreous Proteome and Inflammatory Mediators in Ocular Inflammatory Disease	NCT00331331
Ischemic Stroke∗	Plasma	N/A; Completed	Denmark	N/A	Rheo-Erythrocrine Dysfunction as a Biomarker for RIC Treatment in Acute Ischemic Stroke (ENOS)	NCT04266639
Prostate cancer	Urine	N/A; Completed	N/A	N/A	Clinical Validation of a Urinary Exosome Gene Signature in Men Presenting for Suspicion of Prostate Cancer	NCT02702856
Chronic Kidney Failure; Dialysis Related Complication	N/A	PHASE1 | PHASE2; Completed	Italy	N/A	Effect of Mixed On-line Hemodiafiltration on Circulating Markers of Inflammation and Vascular Dysfunction	NCT03202212
Sleep Apnea; Inflammation; Atherosclerosis	N/A	N/A; Completed	France	N/A	PTP1B Implication in the Vascular Dysfunction Associated With Obstructive Sleep Apnea	NCT04235023
Hypertension	Urine	N/A; Completed	Switzerland	N/A	Phosphate in Blood Pressure Regulation (Phos-RR)	NCT02822131

## 8 Future perspectives: micropuncture exosomes

Reconstructive surgeons have very limited pro-angiogenic treatment options for wound repair. Therefore, they are often required to perform skin grafts or complex vascularized tissue flaps which are morbid and have donor site limitations. Alternatives are warranted and exosome-modulation is at the forefront of regenerative vascularized surgery. The ability to trigger sprouting angiogenesis is vital to the success of both surgical engineering and acellular therapeutic approaches to induce tissue repair. The rate limiting step in this dynamic process requires disruption of the basement membrane of the parent vessel. Membrane degradation is modulated through the accumulation of inflammatory cells (e.g., neutrophils and Mφs), MMPs, growth factors, and cytokines at the target site. In some scenarios, mechanical stress or hypoxic conditions may also initiate the breakdown of the basement membrane. We present the field with a different consideration. Recently, we developed a microsurgical approach, termed micropuncture (MP), that can be used to trigger angiogenesis and rapidly vascularize an adjacently placed hydrogel scaffold (Hancock et al., 2021). MP uses a 60 µm needle, regularly used in microsurgery for its capillary-like size, to purposefully disrupt the targeted macrovascular membrane and rapidly induce microvascular outgrowth. We have shown that this MP-induced vasculature is stable and perfusable for up to a month (Horchler et al., 2024). Our study revealed that angiogenesis within MP groups was stimulated *via* increased scaffold infiltration of ECs (CD31), M1 Mφs (CD86) and M2 Mφs (CD163). These cellular effects were accompanied by increased expression of other notable pro-angiogenic regulators such as VEGFR-2, Tie-2, and Notch1, all of which work dynamically to induce vascular remodeling of the microenvironment (Hancock et al., 2021; Horchler et al., 2024). The significant infiltration of ECs and polarized Mφs suggests that the MP-induced microvasculature is initiated through an interdependent relationship between the cellular modulators and pro-angiogenic molecules of the microenvironment (Shi et al., 2023; Horchler et al., 2024).

As described in Tables 1 and 2, the ideal regenerative exosome platform would be one that is versatile in design, promotes angiogenesis, and facilitates the recruitment of relevant immune cell populations in addition to the deposition of extracellular matrix components. MP, a surgical intervention, stimulates rapid vascularization and enhances immune cell recruitment, two vital elements to the wound healing cascade. Considering these findings, we present the field with an alternative surgical-based exosome platform that could be leveraged to generate multifaceted pro-angiogenic exosomes for regenerative vascularization. We believe the rapid vascularization and cellular recruitment capabilities of *in vivo* MP, may be translated down to the exosomes released from the injured vessels, or in an *in vitro* model, the ECs themselves. Our future studies will delineate the regenerative capacity of these MP-exosomes in inducing vascularization and relevant immune cell infiltration in hopes of establishing the next-generation of bioengineered exosome solutions to ischemia or injury.

## 9 Conclusion

The therapeutic potential of exosome-based platforms in stimulating angiogenesis and wound repair is increasingly evident, particularly in the context of tissue engineering and regenerative vascularization. Exosomes have gained traction within the field of regenerative medicine due to their potential as a regenerative nanotherapeutic with low immunogenicity, increased biocompatibility, storage stability, and controllable dosing. These sEVs exert similar pro-angiogenic and pro-healing effects to their parent cell. We present a comprehensive review of both stem cell-derived and mature cell-derived exosomes in the context of regenerative vascularization and wound repair. An optimal angiogenic exosome platform would possess the ability to recruit polarized M2 reparative macrophages, promote neovascularization within the wound microenvironment, and stimulate timely ECM production and deposition. These pro-angiogenic and regenerative properties are most prominently observed in exosomes derived from adipose derived stem cells, endothelial progenitor cells, and M2 macrophages. However, the clinical translation of these sEVs remains limited due to incomplete mechanistic understanding, variability in isolation methods, and a lack of comprehensive clinical data. Future research in this area should focus on the standardization of exosome production, uncovering the underlying mechanism of action or signaling pathways, in addition to exploring bioengineering approaches to enhance reliability and efficacy of these sEVs. In line with this, we also highlight how materials science will be instrumental to both therapeutic hydrogel-embedded exosome delivery and development of fully acellular pro-angiogenic bioengineered exosomes. Continued research into this exciting and emerging field is warranted, as harnessing the therapeutic potential of pro-angiogenic and pro-healing exosomes will add to the armamentarium for the treatment of hypoxic and ischemic wounds.
